# First Identified Cases of SARS-CoV-2 Variant P.1 in the United States — Minnesota, January 2021

**DOI:** 10.15585/mmwr.mm7010e1

**Published:** 2021-03-12

**Authors:** Melanie J. Firestone, Alexandra J. Lorentz, Stephanie Meyer, Xiong Wang,, Kathryn Como-Sabetti, Sara Vetter, Kirk Smith,, Stacy Holzbauer, Amanda Beaudoin, Jacob Garfin, Kristin Ehresmann, Richard Danila, Ruth Lynfield

**Affiliations:** ^1^Minnesota Department of Health; ^2^Epidemic Intelligence Service, CDC; ^3^Division of State and Local Readiness, Center for Preparedness and Response, CDC.

Since December 2020, the Minnesota Department of Health (MDH) Public Health Laboratory has been receiving 100 specimens per week (50 from each of two clinical partners) with low cycle threshold (Ct) values for routine surveillance for SARS-CoV-2, the virus that causes COVID-19. On January 25, 2021, MDH identified the SARS-CoV-2 variant P.1 in one specimen through this surveillance system using whole genome sequencing, representing the first identified case of this variant in the United States. The P.1 variant was first identified in travelers from Brazil during routine airport screening in Tokyo, Japan, in early January 2021 (*1*). This variant has been associated with increased transmissibility (*2*), and there are concerns that mutations in the spike protein receptor-binding domain might disrupt both vaccine-induced and natural immunity (*3*,*4*). As of February 28, 2021, a total of 10 P.1 cases had been identified in the United States, including the two cases described in this report, followed by one case each in Alaska, Florida, Maryland, and Oklahoma (*5*).

The first Minnesota P.1 variant case was identified in a person who became symptomatic in early January and was hospitalized for 9 days. During the case investigation, the person reported having traveled to southeastern Brazil within the 14 days before symptom onset. The patient’s travel partner, who lived in the same household, also had symptoms of COVID-19 and received a positive SARS-CoV-2 test result after returning. The diagnostic specimen from this household contact was obtained for whole genome sequencing and confirmed to be the P.1 variant. The sequences from both patients were identical and had 15 of the 17 mutations associated with the P.1 variant, including the 10 S-gene mutations (*2*). The Minnesota patients were reinterviewed to obtain information on exposures and close contacts.^†^ This activity was reviewed by CDC and conducted consistent with applicable federal law and policy.^§^

The hospitalized Minnesota patient had interacted with four Minnesota health care facilities. Risk assessments were conducted for 111 health care personnel who provided care, and they were offered testing. No high-risk exposures^¶^ were identified among these health care personnel; 22 (20%) submitted specimens for testing, and no positive test results were reported. The CDC Minneapolis Quarantine Station was notified of potential travel-associated COVID-19 exposures on the arriving international flight and a domestic flight to Minnesota. Because 19 days had passed since the flights, CDC did not initiate a full aircraft contact investigation; however, CDC did obtain information for potentially exposed passengers and notified health departments in their states of residence. In addition to health care personnel, 42 persons in Minnesota who might have had close contact with the patients were notified and offered testing; 20 were tested, and all received negative test results.

The two travel-associated cases of the SARS-CoV-2 variant P.1 in Minnesota represent the first identified occurrences of this variant in the United States. Initial identification of the P.1 variant in Brazilian travelers in Japan and its introduction into Minnesota were identified through routine sequencing, demonstrating the importance of genomic surveillance at state and federal levels to identify variants of concern and to track and prevent their spread (*6*). Genomic surveillance using whole genome sequencing of SARS-CoV-2 specimens is an important public health tool for identifying mutations and monitoring variants of concern (*7*). Identification of the P.1 variant in the United States underscores the importance of community prevention strategies to slow transmission of SARS-CoV-2, including use of well-fitting masks, physical distancing, washing hands, quarantine, testing of persons who have had contact with a person with laboratory-confirmed COVID-19, isolating persons with symptoms of COVID-19 or with diagnosed COVID-19, and adhering to CDC recommendations to delay travel.** In addition, testing should be considered one component of a comprehensive travel risk management strategy. Properly timed testing, both before and after travel, together with self-monitoring for symptoms, a period of self-quarantine after travel, hand hygiene, and physical distancing, are critical elements of this strategy (*8*).^††^

## Footnotes

† Close contact was defined as being within 6 ft of a patient with laboratory-confirmed SARS-CoV-2 infection for a total of ≥15 minutes over a 24-hour period. https://www.cdc.gov/coronavirus/2019-ncov/php/contact-tracing/contact-tracing-plan/contact-tracing.html

§ 45 C.F.R. part 46.102(l)(2), 21 C.F.R. part 56; 42 U.S.C. Sect. 241(d); 5 U.S.C. Sect. 552a; 44 U.S.C. Sect. 3501 et seq.

¶ High-risk exposure among health care providers was defined as having prolonged close contact (≥15 minutes within 6 ft), or contact of any duration during an aerosol-generating procedure, with a person with confirmed COVID-19 or with their secretions or excretions while not wearing appropriate personal protective equipment. https://www.cdc.gov/coronavirus/2019-ncov/hcp/guidance-risk-assesment-hcp.html

**
https://www.cdc.gov/coronavirus/2019-ncov/your-health/need-to-know.html

††
https://www.cdc.gov/coronavirus/2019-ncov/travelers/travel-during-covid19.html
